# Comparative Studies on the Lipid Composition of Blood Plum (*Haematostaphis Barteri*) Pulp and Seed Oils

**DOI:** 10.2174/1874091X01711010094

**Published:** 2017-10-28

**Authors:** Matthew Olaleke Aremu, Hashim Ibrahim, Chrysantus Andrew

**Affiliations:** 1 Department of Chemical Sciences, Federal University Wukari, PMB 1020, Taraba State, Nigeria; 2 Department of Chemistry, Federal University Lafia, PMB 146, Nasarawa State, Nigeria; 3 Department of Chemistry, University of Leicester, Leicester, LE1 7RH, UK

**Keywords:** Fatty acids, Phospholipids, Phytosterols, Physico-chemicals, *Haematostaphis barteri*, Pulp, Seed

## Abstract

**Background:**

Exploring under–utilized plant fruits could be of high significance for food security and nutritional requirements, therefore, it can effectively add to the overall improvement of a nation’s economy. Blood plum (*Haematostaphis barteri*) is a wild edible vegetable and its fruit contains pulp and oily seed which are edible.

**Methods:**

A study was carried out to determine fatty acid profile, phospholipid and phytosterol contents as well as some physicochemical parameters of pulp and seed oils of *Haematostaphis barteri* (popularly known as blood plum) using standard analytical techniques.

**Results:**

The most concentrated fatty acids were palmitic acid (15.34%) < oleic acid (22.31%) < linoleic acid (50.56%) for the pulp oil while that of seed oil were oleic acid (12.34%) < palmitic acid (25.37%) < linoleic acid (38.87%). Arachidic, behenic, lignoceric and palmitoleic acids were all present in small quantities with none of them recording up to 1.0% in either of the samples. Lauric was determined but not detected in the pulp oil. The fatty acid composition of pulp and seed oils contained a healthy mixture of all the types of saturated and unsaturated fatty acids. The value of polyunsaturated/saturated index (P/S) which is associated to the impact on human health was higher in the pulp oil (2.47). Phospatidylinositol had the highest content (17.69 mg/100g) in the pulp oil while the highest content in seed oil was phosphatidylcholine (351.82 mg/100g). The total phytosterols for pulp and seed oils were 17.09 and 436.37 mg/100g, respectively. The results of some physicochemical parameters of pulp and seed oils were colour (light amber yellow and pale yellow), kinematic viscosity (9.15 and 10.62 mm^2^/s), specific gravity (0.92 and 0.92), unsaponifiable matter (1.63 and 2.54%), flash point 29.00 and 295.00^o^C), saponification value (189.40 and 190.22 mg KOH/g), peroxide value (8.15 and 9.20 meq O_2_/kg), iodine value (94.24 and 122.42 mg of I/100 g) and acid value (16.50 and 24.00 mg KOH/g), respectively.

**Conclusion:**

Generally, high percentage PUFA and low value of cholesterol may make either of the sample oils, a good food source on health wise basis. It was also revealed that both sample oils may be developed into a commercial product for use in food products.

## 1 INTRODUCTION

It is a well known fact that nutrition is an issue of both quantity and quality of food that contains essential nutrients for normal body functions. Vegetables and fruits in all their forms ensure an adequate intake of most essential nutrients and vitamins, dietary fibers, and phytochemicals which can supply the much-needed measure of balance diets thereby contributing to solve many of the nutritional problems. They also play an important role in human nutrition and health particularly as sources of vitamin C, niacin, pyridoxine, folic acid, thiamine, minerals and dietary fibre [1]. Scientific evidence shows that frequent consumption of vegetables and fruits can prevent oesophageal, stomach, pancreatic, bladder and cervical cancers and that a diet high in fruits and vegetables could prevent 20% of most types of cancer [2].

Since good nutrition is a basic human right, there is a need for the exploitation of available local resources in order to satisfy the needs of the increasing population in developing countries which is characterized by abject poverty. Studies on wild edible fruits and vegetables indicate that they are good sources of human nutrition, since they contain the essential minerals and vitamins in addition to proteins, fibres, fats and carbohydrate which are very significant for healthy growth and development of human body.

Blood plum (*Haematostaphis barteri*) is a wild edible vegetable belonging to the *Anacardiaceae* family. It is a perennial tree crop which normally grows wildly in the forest and usually among savannah [3]. The tree is found wild in Borno and Adamawa [4], and Taraba States of Nigeria, and it is known as Jinin Kafiri in Hausa language. The leaves of blood plum are used for seasoning soup in some local government areas of Adamawa and Taraba states as well as animal feed. It is also used in nursing the snake bite victims, therefore, the plant bark is also used in treating liver, gall bladder, spleen disorders and jaundice, whereas the wood is used as firewood [5]. The *H. barteri* fruit has oily seed which is edible [4].

Due to the increasing incidence of diet related diseases such as obesity, hypertension and cardiovascular diseases in particular, the study is aimed at analyzing the fatty acid composition, phospholipid and phytosterol contents as well as some physicochemical parameters of pulp and seed of *Haematostaphis barteri,* with a view to providing information regarding effective utilization of the plant’s pulp and seed in various foods and with the possibility of industrial applications.

## 2 MATERIALS AND METHODS

### 2.1 Collection of Samples

The fruits of blood plum (*Haematostaphis barteri*) were purchased from a local market in Zing local government area, Taraba State, Nigeria. The fruits were thoroughly sorted to remove the stones, and the bad ones before washing with tap water. The clean fruits were then transported to the laboratory and proper identification was made by a Taxonomist in the Department of Biological Sciences, Federal University Wukari, Taraba State, Nigeria.

### 2.2 Preparation of Samples

Fifty number clean fruits of *H. barteri* were dried in an oven at 40°C for 5 days in order to separate the pulp from the seed because the pulp is thin and juicy. The pulp was separated from the seed, and freely ground with Kenwood food blender. The seed was also carefully de-shelled using kitchen knife, and the kernel was dried in an oven at 45°C for 36h and ground into powder. Flours of the two samples were separately kept in the refrigerator at -4°C prior to use.

### 2.3 Extraction of Oils

Each oven dried sample of pulp and seed (5g each) of *H. barteri* was extracted for 5h in Soxhlet apparatus with 200 ml of petroleum ether (40-60^0^C boiling range) of Analar grade (British Drug Houses, London). The extraction flask was removed from the heating mantle when it was almost free of petroleum ether, oven dried at 105^0^C for 1h, cooled in a desiccator and used for further analysis [6].

### 2.4 Fatty Acid Analysis

The oil extracted from each sample was converted to the methyl ester using the method described by Adeyeye and Adesina [7]. A 50 mg aliquot of the dried oil was saponified for 5 min at 95°C with 3.4 ml of 0.5 M KOH in dry methanol. The mixture was neutralized by 0.7 M HCl and 3 ml of 14% boron trifloride in methanol was added. The mixture was heated for 5 min at 90°C to achieve complete methylation. The fatty acid methyl esters were analyzed using an HP 6890 gas chromatograph powered with HP Chemistation Rev. a 09.01 (1206) software fitted with a flame ionization detector and a computing integrator. Nitrogen was used as the carrier gas. The column initial temperature was 250°C rising at 5°C/min to a final temperature of 310°C, while the injection port and the detector were maintained at 310°C and 350°C, respectively. A polar (HP INNO Wax) capillary column (30 m × 0.53 mm × 0.25 μm) was used to separate the esters. The peaks were identified by comparison with standard fatty acid methyl esters obtained from Sigma Chemical Co. (St. Louis MO, USA) [8]. However, the quantitative evaluation was carried out on the basis of gas chromatography peak areas of the different methyl esters. The heptadecanoic ester was used to calculate the response factor for fatty acids which was found to be 0.96. Three determinations were made for each sample.

### 2.5 Phospholipid Analysis

The phospholipid content of the pulp and seed oils was determined by gas chromatography (GC). 0.01 g of the extracted fats was added to the test tube. To ensure complete dryness of the oil for phospholipids analysis, the solvent was completely removed by passing the stream of the nitrogen gas on the oil. 0.04 mL of chloroform was added to the content of the tube and it was followed by the addition of 0.10 mL of chromogenic solution. The content of the tube was heated at a temperature of 100°C in a water bath for about 1 min. The content was allowed to cool, 5 mL of the hexane was added and the tube with its content shook gently several times. The solvent and the aqueous layers were recovered and allowed to be separated. The hexane layer was recovered and allowed to be concentrated to 1.0 mL for gas chromatography using flame photometric detector. The conditions for phospholipid analysis include: H.P 5890 powered with HP ChemStation REV. A 09.01 (1206) and split injection ratio of 20:1, nitrogen as carrier gas, inlet temperature 250°C, column type HP5, column dimension: 30 m × 0.25 mm × 0.25 µm, oven program: initial temperature at 50°C, first ramping at 10°C/min for 20 min, maintained for 4 min while second ramping at 15°C/min for 4 min, maintained for 5 min. Detector: PFPD Detector temperature was 300°C, hydrogen pressure being 20 psi, compressor air being 35 psi [8].

### 2.6 Phytosterol Analysis

The phytosterol analysis was done as described by AOAC [6]. The aliquots of the extracted fat were added to the screw - capped test tubes. The samples were saponified at 90°C for 30min, using 3 ml of 10% KOH in ethanol, to which 0.20 mL of benzene was added to ensure miscibility. Deionized water (3 mL) was added and 2 mL of hexane was added in extracting the non – saponifiable materials. Three different extrations, each with 2 mL of hexane were carried out for 1h, 30min and 30min, respectively. The hexane was concentrated to 1 mL in the vial for gas chromatography analysis and 1 μL was injected into the injection pot of GC. The GC conditions of analyses were similar to the GC conditions for methyl esters analyses [8].

### 2.7 Physico-Chemical Analyses

The physico-chemical analyses of the *H. barteri* pulp and seed oils for kinematic viscosity, specific gravity, unsaponifiable matter, flash point, saponification value, peroxide value, iodine value and acid value were carried out according to the methods of AOAC [6]. Three determinations were made for each sample.

### 2.8 Statistical Analysis

The statistical calculations included percentage value, grand mean, standard deviation and relative standard deviation.

## 3 RESULTS AND DISCUSSION

The results of fatty acid composition of *H. barteri* indicate that both the pulp and seed oils have a high content of linoleic acid (C18:2) of 50.56% and 38.87%, respectively (Table **1**). It was also observed that the oils contained significant amount of unsaturated fatty acids of 78.43% and 66.65% for the pulp and seed oils of *H. barteri.* Palmitic acid (C16:0) was found to be predominant saturated fatty acid (SFA) in the oil samples with values of 15.34% (pulp) and 25.37% (seed). The observed values of palmitic acid are in agreement with the reported data of some leguminious plant seeds such as *C. lanatus* (17.71%) and 19.15% for *T. cucumerina* seed oils, and 20.87% for *G. jasminoide* by [9, 10]. respectively. It is one of the most common saturated fatty acid found in cheese, milk, butter, animals and plants and it is an antioxidant, a nematicide used in making soups.

Some studies have indicated the various impacts of SFAs on the human health. It has been concluded that lauric acid (C12:0) as well as myristic acid (C14:0) raise plasma total cholesterol concentrations, due to an increase in low-density lipoprotein (LDL) cholesterol and the rise of both LDL and high-density lipoprotein (HDL) cholesterol concentrations respectively [11]. However, the ratio of total cholesterol to HDL cholesterol is the basic cause of coronary artery diseases than the value of LDL cholesterol [12]. Oils rich in lauric acid (C12:0) decreased the ratio of total to HDL cholesterol. On the other hand, myristic (C14:0) and palmitic acids (C16:0) affected this ratio only little and stearic acid (C18:0) slightly reduced this ratio. Lauric acid was not found as a component of the *H. barteri* pulp oil in the study.

It is observed that the unsaturated fatty acids (linoleic and oleic) account for most of the *H. barteri* pulp and seed oils. It is also noted that linoleic and oleic acids are the major fatty acids in peanut, soy beans and lentil [13]. The pulp oil has the highest content of monounsaturated fatty acids (MUFAs); that is, oleic acid (C18:1) (22.31%). Various studies indicate that a diet rich in oleic acid decreases the development of atherosclerosis and lowers serum cholesterol by diminishing oxidative stress and inflammatory mediators while promoting antioxidant defenses [10]. Davidson *et al.* [14] and Wang *et al.* [15] all reported that the deleterious effects of oleic acid have been attributed to increase in the permeability of both vascular and alveolar epithelium to solute caused by changes in membrane fluidity and increase in intracellular calcium concentration.

Linoleic acid (C18:2) occurs most abundantly in soybean oil, corn oil, sesame oil, peanut oil, grape seed oil and sunflower oil which are leguminous seeds. The amount of linoleic acid (50.55%) and (38.87%) in pulp and seed oils of *H. barteri* (Table **1**) is comparable with that reported for harms seed (47.95%) [16], soy beans (52.0%) [17], boiled tiger nut (46.71%) [8], but lower than that of *Adenopus breviflorus* oil (60.73%) [18], *L. breviflora* oil (61.10%) [19]. Linoleic acid plays a significant role in the skin. In dry skin, it strengthens the lipid barrier of epidermis, protects against transepidermal loss of water and normalises the skin metabolism. Linoleic acid is a natural component of sebum. In persons with acne skin, a decrease in LA content in sebum is observed, which leads to blocked pores and formation of comedos and eczemas. The use of linoleic acid for oily skin and problematic skin care leads to improvement of the work of sebaceous glands, unblocking of pores and decrease in the number of comedos. Moreover, this acid is built in the structure of cell membrane and is also used for production of intercellular cement of the skin. These two processes are possible thanks to the presence of the enzymatic complex in the stratum corneum of epidermis [20]. The seed oil of *H. barteri* contained the highest concentrations of all the fatty acids except for stearic (C18:0), palmitoleic (C16:1), linoleic acid (C18:2) and α - linolenic acids which differ from the pulp oil by 14.23%, 87.20%, 23.11% and 33.04% respectively. The level of RSD ranged from 7.71% in Stearic acid to 88.50% in lignoceric acid (Table **1**).

SD = Standard Deviation; RSD = Relative Standard Deviation; TSFA = Total Saturated Fatty Acid; TMUFA = Total Monounsaturated Fatty Acid; TUFA = Total Unsaturated Fatty Acid; TEFA = Total Essential Fatty Acid; TNEFA = Total Non-Essential Fatty Acid; O/L = Oleic/Linoleic Ratio; TPUFA = Total Polyunsaturated Fatty Acid; n-6/n-3 = linoleic (n-6)/α–linolenic (n-3) Ratio, P/S = Polyunsaturated Fatty Acid/Saturated Fatty Acid.

Table **1** also presents distributions of results into TSFA, TMUFA, TPUFA TUFA, TEFA, TNEFA, oleic to linoleic (O/L) and linoleic to α - linolenic (n6/n3) ratios. It was generally observed that the percentage of both unsaturated and essential fatty acids in pulp oil was higher than that of seed oil, whereas the percentages of saturated and non essential fatty acids were higher in the seed oil sample. TSFA ranged from 21.57% to 33.35% in pulp and seed *H. barteri* oils. These values are higher than the reported values of 9.0% - 12.9% for pinto bean [21] and 17.06% for *B. eurycoma* [16]. High dietary intakes of saturated fatty acids (SFAs) are a risk factor for development of obesity, cardiovascular disease.

The most abundant PUFA was linoleic acid (LA, C18:2, n-6) in all analyzed samples, in the range from 38.87% in seed oil to 50.56% in pulp oil. Similar results of LA predominating have been reported for grape, almond, wheat germ, sesame, pumpkin seed and safflower oil [22]; for peanut, rapeseed and coconut oil [23]; and for hemp oil [22]. The high content of linoleic acid in both oils is an indication that the oil is of high nutritional value for linoleic acid being an essential acid with cholesterol-lowering activity [7].

Recent reports have shown the important impact of polyunsaturated fatty acids (PUFAs) on human health in the prevention of, particularly, cardiovascular disease (CVD), coronary heart disease and cancer; further, inflammatory, thrombotic and autoimmune disease; hypertension; diabetes type two, renal diseases; and rheumatoid arthritis, ulcerative colitis, and Crohn’s disease. Their non-substitutable roles in many biological pathways are crucial [24]. Linolenic acid is n-3 polyunsaturated fatty acid that plays an important role in the regulation of biological functions, prevention and treatment of a great number of human diseases such as heart and inflammatory diseases [25].

Moreover, linoleic acid as reported by Gunstone and Norris [26] usually cures essential fatty acid (EFA) deficiency as it can be neutralized to the required C20 and C22 polyene acids. Essential fatty acid deficiency in human results in abnormal skin conditions such as scaliness and dermatitis, increased water loss, reduced regeneration of tissue and increase susceptibility to infection [26]. Therefore, as the pulp oil contained higher PUFA than the seed oil, it is clear that the cholesterol of the pulp oil could be very low and this will make it very useful for foods preparation to reduce the incidence of heart attack (arteroscleroses) caused by high intake of cholesterol. The TEFA in pulp oil (52.55%) is much higher than TEFA in rice, sorghum, millet, maize and Bambara groundnut [27, 28].

The oleic/linoleic (O/L) acid ratio has been associated with high stability and potentiality of the oil for deep frying fat. The O/L levels were 0.32% in seed and 0.44% in pulp oils of *H. barteri*. These values are lower than that of *Anarcadium occidentale* (12.28%) [29], peanut oil (1.48%) [30], raw tiger nut (2.11%) [14], hence *H. barteri* oils may not be stable compared with soya bean, peanut and groundnut oils. The relationship between the saturated and polyunsaturated fatty acid content is expressed as P/S index. In a diet, it is important the index value be higher than 1, due to the essential character of the linoleic fatty acid (n-6) [31]. Several studies indicate that the P/S relation influences in the level of nutrient metabolization in the body, and as the proportion increases a smaller deposition of lipids [32]. The values of the P/S indexes of the oils studied are shown in Table **1** with values greater than 1.

The total unsaturated fatty acids (TUFA) were 66.65% in seed and 78.43% in pulp oils. The linoleic (n-6) and α–linolenic (n-3) fatty acids have critical roles in the membrane structure and as precursors of eicosanoids, which are potent and highly reactive compounds. Since they compete for the same enzymes and have different biological roles, the balance between the n-6 and n-3 fatty acids in the diet can be of considerable importance [31]. The recommended ratio of linoleic to α–linolenic acids in the diet should be between 5:1 and 10:1 [31]. The ratios of linoleic to α–linolenic of the samples in the present study ranged from 25.40:1 to 29.23. This shows that on a diet based mainly on *H. barteri*; FAO/WHO nutritional requirement will be met. The extracted oil from the pulp sample contained high concentration of MUFA, PUFA, TUFA, TEFA and O/L (%) which differ positively by 0.96%, 21.63%, 15.02%, 23.48% and 27.27%, respectively; from that of the seed oil, while the TSFA and TNEFA (%) of the seed oil sample varies respectively by 54.61% and 26.01% from the pulp sample (Table **1**).

Phospholipids content of *H. barteri* pulp and seed oils are shown in Table **2**. From the result, the seed oil contained the highest concentration of total phospholipids, 771.80 mg/100g as compared to 292.46 mg/100g for pulp oil. Phosphatidycholine (PC) had the highest value of 351.82 mg/100g in seed sample while phosphatidylinositol (PI) showed a greater concentration with the value of 176.69 mg/100g in pulp sample. PC is usually the most abundant phospholipid in animals and plants, often amounting to almost 50% of the total, and as such it is obviously a key building block of membrane bilayers. In particular, it makes up a very high proportion of the outer leaflet of the plasma membrane. It is also said to be the principal phospholipid circulating in plasma, where it is an integral component of the lipoproteins, especially the HDL, as it is regarded as the only phospholipid necessary for lipoprotein assembly and secretion [33-35]. PI is an important lipid, both as a key membrane constituent and as a participant in essential metabolic processes in all plants and animals, both directly and via a number of metabolites. The inositol phospholipids (such as phosphatidylinositol) are the main source of diacylglycerols that serve as signaling molecules in animal and plant cells, trough the action of a family of highly specific enzymes collectively known as phospholipase C. They regulate the activity of a group of at least a dozen related enzymes known as protein kinase C, which in turn control many key cellular functions, including differentiation, proliferation, metabolism and apoptosis. In addition to that, inositol phospholipids appear to have crucial roles in interfacial binding of proteins and in the regulation of proteins at the cell interface [36].

Phosphatidylethanolamine (PE) is usually the second most abundant phospholipid in animal and plant lipids [37]. The PE values were 164.33 mg/100g in seed and 56.74 mg/100g in pulp samples. In animal tissues, phosphatidylethanolamine is especially important in the sarcolemmal membranes of the heart during ischemia, it is involved in secretion of the nascent very-low-density lipoproteins from liver and it has functions in membrane fusion and fission. It also has a functional role in the Ca^2+^- ATPase in that one molecule of PE is bound in a cavity between two trans–membrane helices, acting as a wedge to keep them apart. This is displaced when Ca^2+^ is bound to the enzyme [38]

Phosphatidyserine (PS) and lysophosphatidycholine (LPC) were the minor phospholipids with concentrations ranging between 33.66 to 38.37 mg/100g and 2.52e^-2^ to 9.85 mg/100g, respectively. In addition to its function as a component of cellular membranes and as a precursor for other phospholipids, PS is an essential cofactor that binds to and activates a large number of proteins, especially those with signalling activities [39]. LPC (which is mostly found in trace amounts in most tissues) has pro-inflammatory properties and it is known to be a pathological component of oxidized lipoproteins (LDL) in plasma and of atherosclerotic lesions; it has been shown to promote demyelination in the nervous system. Recently, LPC has been found to have some functions in cell signalling, and specific receptors (coupled to G proteins) have been identified. It activates the specific phospholipase C that releases diacylglycerols and inositol triphosphate with resultant increases in intracellular Ca^2+^ and activation of protein kinase C. LPC also activates the mitogen-activated protein kinase in certain cell types [40]. The RSD ranged from 6.55% in phosphatidylserine to 99.04% in lysophosphatidylcholine.

The most abundant plant phytosterols are sitosterol, campesterol and stigmasterol [41]. The total phytosterol concentrations of the studied samples were 17.09 and 436.37 mg/100g for pulp and seed oils, respectively (Table **3**). Seed sample was more concentrated in all the plant sterols than the pulp oil. The RSD varied from 24.90 to 98.46.

The daily dietary intake of plant sterols differs among populations, but is typically between 160-400 mg. Those eating a vegetarian diet may eat up to 750 mg/day, which would provide some degree of cholesterol lowering [42]. Therefore, oil extracted from the seed of *H. barteri* sample will be a very good source of dietary phytosterols. In addition to their cholesterol lowering properties, phytosterols possess anti-cancer, anti-inflammatory, anti-atherogenicity, and anti-oxidation activities, and should thus be of clinical importance, even for those individuals without elevated LDL cholesterol [43].

The results of the physicochemical analysis of the *H. barteri* pulp and seed oils are presented in Table **4**. The iodine, acidity, peroxide and saponification values are the major characterization parameters for oil quality. Peroxide value (PV) is a measure of oxidation during storage and the freshness of lipid matrix. It is a useful indicator of the early stages of rancidity occurring under mild condition and also, a measure of the primary lipid oxidation products. One of the most important parameters that influence lipid oxidation is the degree of unsaturation of its fatty acids. When double bonds of unsaturated fats are oxidized, peroxides are among the oxidation products formed. Peroxide values of all the oil samples were in agreement with the maximum Codex standard peroxide value (10 meq O_2_/kg) for vegetable oil deterioration. The *H. barteri* oils have significantly high peroxide values (8.15 meq O_2_/kg for pulp and 9.20 meq O_2_/kg for seed) and hence high degree of unsaturation. This observation suggests that *H. barteri* oils have high content of unsaturated fatty acids, linoleic (C18:2) and oleic acid (C18:1), which are responsible for oxidative rancidity.

Acid value is a measure of the free fatty acids in oil. The higher the acid value found, the higher the level of free fatty acids which translates into decreased oil quality. Acceptable levels for all oil samples should be below 0.6 mg KOH/g (measured in potassium hydroxide per gram) [44]. Among the oil samples studied, the seed oil has highest (24.00 mg KOH/g) acid value which indicates high free fatty acids and leads to a tendency to become rancid (off-flavor). Acid value is also used as an indicator for edibility of an oil and suitability for use in the paint and soap industries [45]. High acid values in the oils (16.50mg KOH/g and 24.00mg KOH/g) for pulp and seed samples, respectively showed that the oils may not be suitable for use in cooking (edibility), but however, be useful for production of paints, liquid soap and shampoos [45]. Acid value of the oil suitable for edible purpose should not exceed 4 mg KOH/g [46].

Iodine index is a property of the unsaturation of fatty acids or its esters. Lipids with unsaturated fatty acids (containing one or more double bonds) are easily assimilated and broken down to produce calorific energy than saturated fatty acids. However, when the iodine value becomes too high, the stability of the oil reduces because it is more likely to undergo oxidation. Oils with iodine value above 125mg of I/100g are classified as drying oils; those with iodine value 110 - 125mg of I/100g are classified as semidrying oils. Those with iodine value less than 110 are considered as nondrying oil [45]. The iodine values of the extracted oils were 94.24 and 122.42 g I_2_/100g for pulp and seed, respectively. The value is high for seed sample, indicating that it is semidrying oil. Thus, the oil will not attract high interest in the paint and coatings industry unless it undergoes dehydration before use. The differences in iodine values between pulp and seed oil samples may be due to the different fatty acid compositions.

Saponification value (SV) is an indicator of the average molecular weight and, hence, chain length. It is inversely proportional to the molecular weight of the lipid. The SV values of the studied oils were 189.40 mg KOH/g and 190.22 mg KOH/g for pulp and seed samples, respectively. The results are in good agreement with Codex standard for cotton oil (189 - 198 mg KOH/g), soybean oil (189 - 195 mg KOH/g), corn oil (187 - 195 mg KOH/g) and peanut oil (187 - 196 mg KOH/g) [46], but lower than the 246.60 for African pear seed oil [47], 227.49 for groundnut seed oil [47] and 224.40 for shea butter tree seed oil [48]. Oil fractions with saponification values of 200 mg KOH/g and above, had been reported to possess low molecular weight fatty acids [45].

The unsaponifiable matter of the oil samples were 1.63% (pulp) and 2.54% (seed). These values are low especially in the pulp oil, which indicate that the oil sample is pure because most fats and oils of normal purity contained less than 2% of unsaponifiable matter [49]. Specific gravity of the extracted oils was 0.92 gcm^–3^ in both pulp and seed samples. The result indicates that the studied oils are less dense than water and therefore would be useful in cream production as it will make the oils flow and spread easily on the skin. The low specific gravity of *H. barteri* oil implies good shelf-life characteristics. The viscosities of the investigated oils were 9.15 and 10.62 in pulp and seed oils, respectively. Oils with low viscosity value indicate that they are light and so probably highly unsaturated. The high value might be as a result of suspended particles still present in the crude oil sample. Kinematic viscosity increases with FA chain length and with increasing degree of saturation of either the fatty acid or alcohol moiety in a fatty ester [50].

Flash point is the lowest temperature at which a liquid can form an ignitable mixture in air near the surface of the liquid. It has value of 290.00 (pulp) and 295.00 (seed) *H. barteri* oils. The lower the flash point, the easier it is to ignite the material. The extracted oils were light amber yellow and pale yellow colour for pulp and seed oils of *H. barteri* samples, respectively. The relative standard deviation (RSD) ranged from 0.00% in specific gravity to 18.52 in acid value.

## CONCLUSION

The study presented data on the concentrations of fatty acid, phospholipid and phytosterol compositions of *Haematostaphis barteri* pulp and seed oils. Some physico–chemical parameters were also presented. There was a clear indication that the pulp and seed oils contained a high level of polyunsaturated fatty acids, making them a healthy low fat food. It was also revealed that both sample oils may be developed into a commercial product for use in food products.

## Figures and Tables

**Table 1:** Fatty acids composition (%) of oils extracted from pulp and seed *H. barteri*.

**Fatty acid**	***^a^H. barteri***		**Mean**	**SD**	**RSD**
	**Pulp**	**Seed**			
C12:0	0.0000±0.00	1.2113±0.10	0.6057	0.43	70.99
C14:0	0.2663±0.10	1.2167±0.10	0.7415	0.48	64.73
C16:0	15.3409±0.20	25.3689±0.10	20.3549	5.01	24.61
C18:0	5.8654±0.10	5.0308±0.10	5.4481	0.42	7.71
C20:0	0.0384±0.10	0.0652±0.20	0.0518	0.01	19.31
C22:0	0.0354±0.10	0.0601±0.10	0.0478	0.01	20.92
C24:0	0.0238±0.01	0.4055±0.20	0.2147	0.19	88.50
C16:1 (Cis–9)	0.5686±0.01	0.0728±0.11	0.3207	0.25	77.95
C18:1 (Cis–6)	2.0579±0.05	12.1853±0.12	7.1216	5.06	71.05
C18:1 (Cis–9)	22.3085±0.01	12.3375±0.10	17.3230	4.99	28.81
C20:1 (Cis–11)	0.0644±0.02	0.1094±0.10	0.0869	0.02	23.01
C22:1 (Cis–13)	0.0516±0.02	0.0877±0.01	0.0697	0.02	28.69
C24:1 (Cis–15)	0.0044±0.02	0.0074±0.01	0.0059	0.002	33.98
C18:1 (Trans–6)	0.0138±0.50	0.0234±0.10	0.0186	0.01	53.76
C18:1 (Trans–9)	0.0012±0.01	0.0021±0.03	0.0017	0.001	58.82
C18:2 (Cis–9,12)	50.5556±0.02	38.8713±0.00	44.7135	5.84	13.06
C18:2 (Trans–9,12)	0.0162±0.01	0.0275±0.10	0.0219	0.01	45.66
C20:2 (Cis–11,14)	0.0054±0.03	0.0092±0.00	0.0073	0.002	0.00
C22:2 (Cis–13,16)	0.0247±0.01	0.0419±0.01	0.0333	0.01	30.03
C18:3 (Cis–6,9,12)	0.6529±0.01	1.3404±0.01	0.9967	0.34	34.11
C18:3 (Cis–9,12,15)	1.9921±0.01	1.3340±0.10	1.6631	0.33	19.84
C20:3 (Cis–8,11,14)	0.0448±0.01	0.0761±0.01	0.0605	0.02	33.06
C20:3 (Cis–11,14,17)	0.0235±0.02	0.0399±0.01	0.0317	0.01	31.55
C20:5 (Cis–5,8,11,14,17)	0.0259±0.01	0.0440±0.01	0.0350	0.01	28.57
C20:6 (Cis–4,7,10,13,16,19)	0.0186±0.03	0.0315±0.02	0.0251	0.01	39.84
TSFA	21.57	33.35			
TSFA%	21.57	33.35			
TMUFA	25.07	24.83			
TPUFA	53.36	41.82			
TUFA	78.43	66.65			
TUFA%	78.43	66.65			
TEFA%	52.55	40.21			
TNEFA	47.45	59.79			
O/L Ratio	0.44	0.32			
P/S Ratio	2.47	1.25			
n-6/ n-3 Ratio	25.41	29.23			

**^a^**Values are means ± standard deviations of three determinations

**Table 2:** Phospholipid levels (mg/100g) of oils extracted from *H. barteri*.

**Phospholipids**	***^a^H. barteri***		**Mean**	**SD**	**RSD**
	**Pulp**	**Seed**			
Phosphatidylethanolamine	56.74±0.50	164.33±0.10	110.54	53.80	48.67
Phosphatidylcholine	25.34±0.15	351.82±0.50	188.58	163.24	86.56
Phosphatidylserine	33.66±0.10	38.37±0.10	36.02	2.36	6.55
Lysophosphatidylcholine	2.52 e^-2^±0.20	9.84±0.20	4.94	4.91	99.44
Phosphatidylinositol	176.69±0.10	207.44±0.02	192.07	15.38	8.00
Total	292.46±0.20	771.80±0.10			

**^a^** Values are means ± standard deviations of three determinations SD = Standard Deviation; RSD = Relative Standard Deviation

**Table 3:** Phytosterols levels (mg/100g) of oils extracted from *H. barteri*.

**Sterols**	***^a^H. barteri***		**Mean**	**SD**	**RSD**
	**Pulp**	**Seed**			
Cholesterol	9.78 e^-5^±0.01	1.29 e^-2^±0.02	6.50 e^-3^	6.40 e^-3^	98.46
Cholestanol	1.99 e^-5^±0.01	1.46 e^-3^±0.00	7.40 e^-4^	7.20 e^-4^	97.33
Ergosterol	1.85 e^-3^±0.10	3.08 e^-3^±0.05	2.47 e^-3^	6.15 e^-4^	24.90
Campesterol	1.63±0.01	42.16±0.01	21.90	20.27	92.56
Stigmesterol	3.40±0.10	16.57±0.02	9.99	6.59	65.97
5-Avenasterol	4.00 e^-1^±0.10	15.93±0.10	8.17	7.77	95.04
Sitosterol	11.66±0.01	361.69±0.01	186.68	175.02	93.75
Total	17.09	436.37			

**^a^**Values are means ± standard deviations of three determinations SD = Standard Deviation; RSD = Relative Standard Deviation

**Table 4:** Physicochemical parameters of oils extracted from *H. barteri*.

**Parameters**	***^a^H. barteri***		**Mean**	**SD**	**RSD**
	**Pulp**	**Seed**			
Kinematic viscosity (mm^2^/s) at 100°C	9.15±0.10	10.62±0.20	9.89	0.74	7.48
Specific gravity (g cm^–3^)	0.92±0.10	0.92±0.10	0.92	0.00	0.00
Unsaponifiable matter (%)	1.63±0.01	2.54±0.20	2.09	0.46	22.01
Flash point (°C)	290.00±0.01	295.00±0.10	292.50	2.50	0.85
Saponification value (mg KOH/g)	189.40±0.01	190.22±0.10	189.81	0.41	0.22
Peroxide value (meq O_2_/kg)	8.15±0.00	9.20±0.10	8.68	0.53	6.11
Iodine value (mg of I/100 g)	94.24±0.10	122.42±0.10	108.33	14.09	13.01
Acid value (mg KOH/g)	16.50±0.05	24.00±0.01	20.25	3.75	18.52
Colour	LAY	PY			

**^a^**Values are means ± standard deviations of three determinations LAY = Light Amber Yellow, PY = Pale Yellow, SD = Standard Deviation, RSD = Relative Standard Deviation

## References

[1] Wargovich M.J. (2000). Anticancer properties of fruits and vegetables.. Hortic. Sci..

[2] Crawford P.B., Obarzanek E., Morrison J., Sabry Z.I. (1994). Comparative advantage of 3-day food records over 24-hour recall and 5-day food frequency validated by observation of 9- and 10-year-old girls.. J. Am. Diet. Assoc..

[3] Aremu M.O., Oko O.J., Ibrahim H., Basu S.K., Andrew C., Ortutu S.C. (2015). Compositional evaluation of pulp and seed of blood plum (*Haematostaphis barteri*), a wild tree found in Taraba State, Nigeria.. Adv. Life Sci. Technol..

[4] Bokhari M.H., Ahmed M.J. (1979). Food plants in Borno State Nigeria..

[5] Ghazanfar S.A. (1989). Savannah plants, an illustrated guide.

[6] AOAC (2005). Official Methods of Analysis..

[7] Adeyeye E.I., Adesina A.J. (2015). Lipid composition of the brains of she-goat and castrated goat consumed in Ekiti State, Nigeria.. Bangladesh J. Sci. Ind. Res..

[8] Aremu M.O., Ibrahim H., Aremu S.O. (2016). Lipid composition of black variety of raw and boiled tigernut (*Cyperus esculentus L.*) grown in North – East Nigeria.. Pak. J. Nutr..

[9] Olonisakin A., Adei-Edeh P.O., Idok R. (2010). Oil quality characteristics of *Cultivars sinnensis* and *Citrullus lanatus* seed oils.. J. Chem. Soc. Nig..

[10] Obuzor G.U., Nwaokolo M.I., Characterization of the fatty acids of Nigerian (Port Harcourt) grown *Gardenia jasminoide* flower (Anachem in Press), 2010

[11] Denke M.A., Grundy S.M. (1992). Comparison of effects of lauric acid and palmitic acid on plasma lipids and lipoproteins.. Am. J. Clin. Nutr..

[12] Mensink R.P., Zock P.L., Kester A.D., Katan M.B. (2003). Effects of dietary fatty acids and carbohydrates on the ratio of serum total to HDL cholesterol and on serum lipids and apolipoproteins: a meta-analysis of 60 controlled trials.. Am. J. Clin. Nutr..

[13] Olaofe D., Ogungbenle H.N., Akhadelor B.E., Idris A.O., Omojola O.V., Omotehinse O.T., Ogunbodede O.A. (2012). Physicochemical and fatty acids composition of oils from some legume seeds. *Int. J. Bio. Pharm.*. Allied Sci..

[14] Davidson K.G., Bersten A.D., Barr H.A., Dowling K.D., Nicholas T.E., Doyle I.R. (2000). Lung function, permeability, and surfactant composition in oleic acid-induced acute lung injury in rats.. Am. J. Physiol. Lung Cell. Mol. Physiol..

[15] Vadász I., Morty R.E., Kohstall M.G., Olschewski A., Grimminger F., Seeger W., Ghofrani H.A. (2005). Oleic acid inhibits alveolar fluid reabsorption: a role in acute respiratory distress syndrome?. Am. J. Respir. Crit. Care Med..

[16] Ajayi F.A., Aremu M.O., Mohammed Y., Madu P.C., Atolaiye B.O., Audu S.S., Opaluwa O.D. (2014). Effect of processing on fatty acid and phospholipid compositions of Harms (*Brachystegia eurycoma*) seed grown in Nigeria.. Chem. Process Eng. Res..

[17] Paul A.A., Southgate D.A. (1985). McCance and Widdowsen’s “The Composition of Foods”..

[18] Oshodi A.A. (1996). Amino acid and fatty acid compositions of Adenopus breviflorus benth seed.. Int. J. Food Sci. Nutr..

[19] Essien E.E., Udo I.I., Ogunwande I.A. (2013). Physicochemical properties, fatty acids composition and antioxidant activity of some cucurbits seed oils.. IJBPAS.

[20] Zielińska P., Nowak I. (2014). Fatty acids in vegetable oils and their importance in cosmetic industry.. CHEMI.

[21] Audu S.S., Aremu M.O., Lajide L. (2011). Effect of procession on fatty acid composition of pinto bean (*Phaseolus vulgaris* L.) seeds.. Int. J. Chem. Sci..

[22] Rajah K.K. (2002). Fats in Food Technology..

[23] Kostik V., Memeti S., Bauer B. (2013). Fatty acid composition of edible oils and fats.. J. Hyg. Eng. Des..

[24] Abedi E., Sahari M.A. (2014). Long-chain polyunsaturated fatty acid sources and evaluation of their nutritional and functional properties.. Food Sci. Nutr..

[25] Shapiro H. (2003). Could n-3 polyunsaturated fatty acids reduce pathological pain by direct actions on the nervous system?. Prostaglandins Leukot. Essent. Fatty Acids.

[26] Gunstone F.D., Norris F.A. (1983). Lipids in Food Chemistry, Biochemistry and Technology..

[27] Aremu M.O., Mamman S., Olonisakin A. (2013). Evaluation of fatty acids and physicochemical characteristics of six varieties of bambara groundnut (*Vigna subterranea* L. Verdc) seed oils.. La Rivista Italiana Delle Sostanze.

[28] Adeyeye A., Ajewole K. (1992). Chemical composition and fatty acid profiles of cereals in Nigeria.. Food Chem..

[29] Aremu M.O., Ogunlade I., Olonisakin A. (2007). Fatty acid and amino acid composition of cashew nut (*Anarcadium occidentale*) protein concentrate.. Pak. J. Nutr..

[30] Branch W.D., Nakayama T., Chennan M.S. (1990). Fatty acid rariation among US runner type peanut cultivars.. J. Am. Oil Chem. Soc..

[31] WHO/FAO (1994). Fats and Oils in Human Nutrition (Report of a Joint Expert Consultation), FAO Food and Nutrition Paper 57..

[32] Lawton C.L., Delargy H.J., Brockman J., Smith F.C., Blundell J.E. (2000). The degree of saturation of fatty acids influences post-ingestive satiety.. Br. J. Nutr..

[33] Cole L.K., Vance J.E., Vance D.E. (2012). Phosphatidylcholine biosynthesis and lipoprotein metabolism.. Biochim. Biophys. Acta.

[34] de Kroon A.I. (2007). Metabolism of phosphatidylcholine and its implications for lipid acyl chain composition in *Saccharomyces cerevisiae*.. Biochim. Biophys. Acta.

[35] Lagace T.A., Ridgway N.D. (2013). The role of phospholipids in the biological activity and structure of the endoplasmic reticulum.. Biochim. Biophys. Acta.

[36] Michell R.H. (2013). Inositol lipids: from an archaeal origin to phosphatidylinositol 3,5-bisphosphate faults in human disease.. FEBS J..

[37] Viel G., Boscolo-Berto R., Cecchetto G., Fais P., Nalesso A., Ferrara S.D. (2012). Phosphatidylethanol in blood as a marker of chronic alcohol use: a systematic review and meta-analysis.. Int. J. Mol. Sci..

[38] Dowhan W. (2009). Molecular genetic approaches to defining lipid function.. J. Lipid Res..

[39] Mozzi R., Buratta S., Goracci G. (2003). Metabolism and functions of phosphatidylserine in mammalian brain.. Neurochem. Res..

[40] Jackson S.K., Abate W., Tonks A.J. (2008). Lysophospholipid acyltransferases: novel potential regulators of the inflammatory response and target for new drug discovery.. Pharmacol. Ther..

[41] Moreau R.A., Whitaker B.D., Hicks K.B. (2002). Phytosterols, phytostanols, and their conjugates in foods: structural diversity, quantitative analysis, and health-promoting uses.. Prog. Lipid Res..

[42] Piironen V., Lindsay D.G., Miettinen T.A. (2000). Plant sterols: biosynthesis, biological function and their importance to human nutrition.. J. Sci. Food Agric..

[43] Berger A., Jones P.J., Abumweis S.S. (2004). Plant sterols: factors affecting their efficacy and safety as functional food ingredients.. Lipids Health Dis..

[44] (2003). AOCS. American Oil Chemists Society, Champaign, IL..

[45] Aremu M.O., Olaofe O., Akintayo E.T. (2006). Chemical composition and physicochemical characteristics of two varieties of bambara groundnut (*Vigna subterrenea*) flours.. J. Appl. Sci. (Faisalabad).

[46] Codex Alimentarius Commission. Graisses et huiles vegetables, division 11, version Abregee FAO/WHO. Codex Stan 20-1981, 23-1981 (1993).

[47] Amos-Tautua B.M., Onigbinde A.O. (2013). Physicochemical properties and fatty acid profiles of crude oil extracts from three vegetable seeds.. Pak. J. Nutr..

[48] Raimi M.M., Adegoke B.M., Afolabi O. (2014). Nutritional composition of seed and physicochemical properties of seed oil of *Vitellaria paradoxa.*. Sci. Res. J..

[49] Pearson D., *The chemical analysis of foods*. 7^th^ Ed. Church Livingstone Longman group limited. **1991**, pp. 493-494

[50] Knothe G., Matheaus A.C., Ryan T.W. (2003). Cetane numbers of branched and straight-chain fatty esters determined in an ignition quality tester.. Fue.

